# KU-0060648 inhibits hepatocellular carcinoma cells through DNA-PKcs-dependent and DNA-PKcs-independent mechanisms

**DOI:** 10.18632/oncotarget.7742

**Published:** 2016-02-26

**Authors:** Min-Bin Chen, Zhen-Tao Zhou, Lan Yang, Mu-Xin Wei, Min Tang, Ting-Yan Ruan, Jun-Ying Xu, Xiao-zhong Zhou, Gang Chen, Pei-Hua Lu

**Affiliations:** ^1^ Department of Oncology, Kunshan First People's Hospital Affiliated to Jiangsu University, Kunshan 215300, China; ^2^ Department of Orthopedics, the Second Affiliated Hospital of Soochow University, Suzhou 215000, China; ^3^ Department of Breast Surgery, the Third Affiliated Hospital of Soochow University, Changzhou 213003, China; ^4^ Department of Traditional Chinese Medicine, First Affiliated Hospital of Nanjing Medical University, Nanjing 210029, China; ^5^ Department of Medical Oncology, Wuxi People's Hospital Affiliated to Nanjing Medical University, Wuxi 214023, China; ^6^ Department of Neurosurgery, the First Affiliated Hospital of Soochow University, Suzhou 215006, China

**Keywords:** hepatocellular carcinoma (HCC), DNA-PKcs, KU-0060648, PI3K-AKT-mTOR signaling, miRNA

## Abstract

Here we tested anti-tumor activity of KU-0060648 in preclinical hepatocellular carcinoma (HCC) models. Our results demonstrated that KU-0060648 was anti-proliferative and pro-apoptotic in established (HepG2, Huh-7 and KYN-2 lines) and primary human HCC cells, but was non-cytotoxic to non-cancerous HL-7702 hepatocytes. DNA-PKcs (DNA-activated protein kinase catalytic subunit) is an important but not exclusive target of KU-0060648. DNA-PKcs knockdown or dominant negative mutation inhibited HCC cell proliferation. On the other hand, overexpression of wild-type DNA-PKcs enhanced HepG2 cell proliferation. Importantly, KU-0060648 was still cytotoxic to DNA-PKcs-silenced or -mutated HepG2 cells, although its activity in these cells was relatively weak. Further studies showed that KU-0060648 inhibited PI3K-AKT-mTOR activation, independent of DNA-PKcs. Introduction of constitutively-active AKT1 (CA-AKT1) restored AKT-mTOR activation after KU-0060648 treatment in HepG2 cells, and alleviated subsequent cytotoxicity. *In vivo*, intraperitoneal (*i.p.*) injection of KU-0060648 significantly inhibited HepG2 xenograft growth in nude mice. AKT-mTOR activation was also inhibited in xenografted tumors. Finally, we showed that DNA-PKcs expression was significantly upregulated in human HCC tissues. Yet miRNA-101, an anti-DNA-PKcs miRNA, was downregulated. Over-expression of miR-101 in HepG2 cells inhibited DNA-PKcs expression and cell proliferation. Together, these results indicate that KU-0060648 inhibits HCC cells through DNA-PKcs-dependent and -independent mechanisms.

## INTRODUCTION

Hepatocellular carcinoma (HCC) is the most common primary liver malignancy in human [1, 2]. Each year, half-million or more people will be diagnosed with this devastating disease. Its incidence has been steadily increasing [1]. Many HCC patients are diagnosed at late or advanced stages with poor prognosis [3]. HCC cells are extremely resistant to almost all conventional chemotherapeutic drugs [4]. Therefore, oncologists are testing novel and molecularly-targeted agents for HCC [3-5].

DNA activated protein kinase (DNA-PK) is a multi-protein complex mainly composed of the 460-kDa catalytic subunit (DNA-PKcs), and the Ku hetero-dimer (Ku-70 and Ku-80) [6, 7]. DNA-PKcs is a phosphatidylinositol-3-kinase (PI3K)-like protein kinase (PIKK) that could be phosphorylated and activated upon DNA damages [8, 9]. Recently, groups have studied the expression and function of DNA-PKcs in several human cancers. These studies proposed that DNA-PKcs is positively involved in cancer initiation, progression, and apoptosis-resistance [10-16]. Therefore, DNA-PKcs represents a novel and critical oncotarget [10-16].

Existing evidences have shown that DNA-PKcs is overexpressed and/or over-activated in multiple human cancers [15]. Inhibition, downregulation or mutation of DNA-PKcs was shown to inhibit cancer cells [10-16]. Meanwhile, DNA-PKcs silence could sensitize cancer cells to radiation and chemotherapy (*i.e.* etoposide and doxorubicin) [12, 13, 16-18]. In the current study, we show that KU-0060648, a recently-developed water-soluble DNA-PKcs inhibitor [12, 19, 20], exerts potent anti-tumor activity in preclinical HCC models.

## RESULTS

### KU-0060648 inhibits HCC cell proliferation

**Figure 1:**
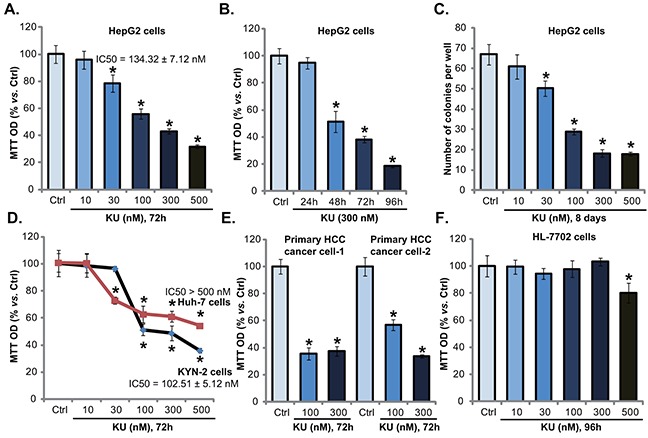
KU-0060648 inhibits HCC cell proliferation HepG2 **A-C.** Huh-7 **D.** and KYN-2 (D) HCC cells, as well as the primary human HCC cells **E.** line-1/-2) and HL-7702 human hepatocytes **F.** were either left untreated (“Ctrl”, same for all figures), or treated with applied concentrations of KU-0060648 (“KU”, 30-500 nM), cells were then cultured for indicated time. Cell proliferation was tested by MTT assay (A and B, D-F) or clonogenicity assay (C). IC-50 was calculated by the SPSS software (A and D). Experiments in this figure were repeated four times, with similar results obtained. n=5 for each repeat. Bars stand for mean ± SD * *p* < 0.05 vs. group “Ctrl”.

To test the potential role of KU-0060648 on HCC cells, HepG2 cells were treated with applied concentrations of KU-0060648. MTT assay results in Figure 1A demonstrated that KU-0060648 dose-dependently inhibited HepG2 cell proliferation, with IC50 = 134.32 ± 7.12 nM. Proliferation inhibition by KU-0060648 in HepG2 cells was also confirmed by results from the [H^3^] Thymidine incorporation assay (Supplementary Figure S1A). Meanwhile, KU-0060648 (at 300 nM) also showed a time-dependent effect in inhibiting HepG2 cells (Figure 1B). Further, the clonogenicity assay results in Figure 1C again demonstrated the anti-proliferative activity by KU-0060648. The number of viable HepG2 colonies was significantly decreased following applied KU-0060648 (30-500 nM) treatment (Figure 1C). Notably, KU-0060648 exerted similar anti-proliferative effect in two other human HCC cell lines: Huh-7 and KYN-2 (Figure 1D and Supplementary Figure S1B).

The potential activity of KU-0060648 in primary human HCC cells was also tested. Using the method described, we successfully cultured two primary human HCC cell lines. These cells were treated with KU-0060648. Results of MTT assay (Figure 1E) and [H^3^] Thymidine incorporation assay (Supplementary Figure S1B) demonstrated clearly that KU-0060648 inhibited primary HCC cell proliferation. Significantly, same KU-0060648 treatment was general safe to non-cancerous HL-7702 human hepatocytes (Figure 1F). Only exception was KU-0060648 at 500 nM, which only slightly inhibited HL-7702 cell proliferation (Figure 1F). One reason could be that HL-7702 hepatocytes express very low level of DNA-PKcs, as compared to primary HCC cells (Supplementary Figure S1C). Further, MTT assay results showed that KU-0060648 was mostly ineffective to the proliferation of two different types of non-cancerous cells, including the human peripheral blood mononuclear cells (PBMCs) and primary human skin fibroblasts (HSFs) (Supplementary Figure S1D). Note that these non-cancerous cells grew much slower than primary and established (HepG2) HCC cells (Supplementary Figure S1E). Together, these results indicate a selective and potent anti-proliferative activity by KU-0060648 against HCC cells.

### KU-0060648 induces caspase-dependent HCC cell apoptotic death

**Figure 2:**
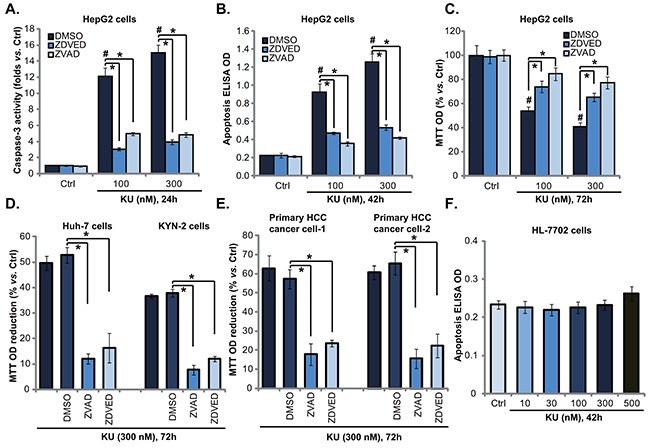
KU-0060648 induces HCC cell apoptotic death HepG2 **A-C.** Huh-7 **D.** and KYN-2 (D) HCC cells, as well as the primary human HCC cells **E.** line-1/-2) and HL-7702 human hepatocytes **F.** pretreated with or without z-DEVD-fmk (“ZDEVD”, 40 μM) or z-VAD-fmk (“ZVAD”, 40 μM) for 1 h, were treated with applied concentrations of KU-0060648 (“KU”), cells were then cultured for indicated time, cell apoptosis was examined by the caspase-3 activity assay (A) or the Histone DNA apoptosis ELISA assay (B and F). Cell proliferation was tested by MTT assay (C-E). Experiments in this figure were repeated four times, with similar results obtained. n=5 for each repeat. Bars stand for mean ± SD ^#^*p* < 0.05 vs. group “Ctrl”. *p < 0.05.

The results above demonstrated that KU-0060648 exerted potent anti-proliferative activity against human HCC cells. We next wanted to know if apoptosis activation was occurred. Two independent assays, including the caspase-3 activity assay and the histone DNA apoptosis ELISA assay [21, 24], were performed. Results from both assays showed that KU-0060648 at 100 and 300 nM induced significant apoptosis activation in HepG2 cells (Figure 2A and 2B). The caspase-3 activity and the apoptosis ELISA OD were both increased following KU-0060648 treatment (Figure 2A and 2B). The caspae-3 specific inhibitor z-DEVD-fmk and the general caspase inhibitor z-VAD-fmk largely inhibited KU-0060648-induced apoptosis activation in HepG2 cells (Figure 2A and 2B). Importantly, KU-0060648-induced anti-HepG2 cell activity, evidenced by MTT OD reduction, was significantly attenuated with pretreatment of the two caspase inhibitors (Figure 2C).

Further studies showed that the caspase inhibitors (z-DEVD-fmk and z-VAD-fmk) dramatically inhibited KU-0060648-induced growth inhibition (MTT OD reduction) in Huh-7 and KYN-2 cells (Figure 2D). Further, z-DEVD-fmk and z-VAD-fmk also protected primary human HCC cells from KU-0060648 (Figure 2E). Once again, no significant apoptosis was noticed in KU-0060648-treated human HL-7702 hepatocytes (Figure 2F). Together, these results demonstrate that KU-0060648 induces caspase-dependent HCC cell apoptotic death.

### DNA-PKcs is an important but not exclusive target of KU-0060648 in HCC cells

**Figure 3:**
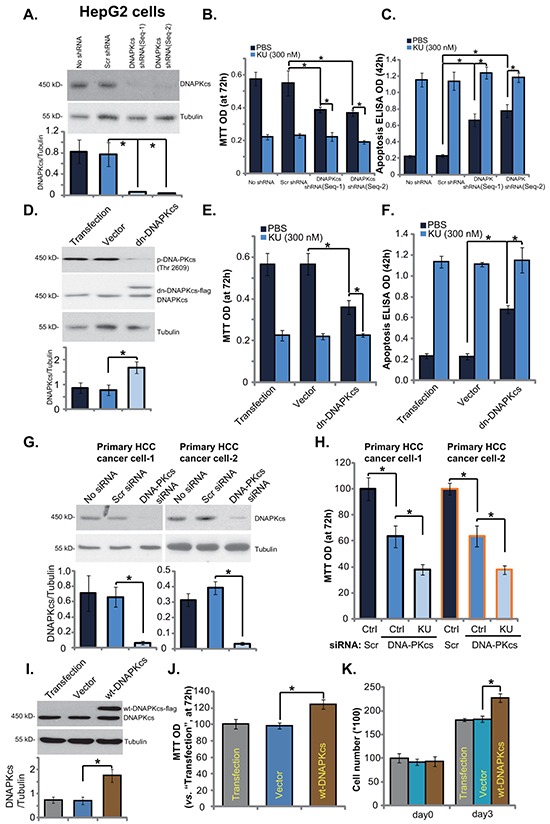
DNA-PKcs is an important but not exclusive target of KU-0060648 in HCC cells Same amount (5 × 10^4^ cells/dish) of stable HepG2 cells expressing scramble control shRNA (“Scr shRNA”), DNA-PKcs shRNA (Seq-1) or DNA-PKcs shRNA (Seq-2), as well as the parental HepG2 cells (“No shRNA”), treated with KU-0060648 (“KU”, 300 nM) or vehicle (PBS) for applied time, were cultured for indicated time. Expressions of DNA-PKcs and tubulin (loading control) were tested by Western blotting **A**. Cell growth was tested by MTT assay **B.** Cell apoptosis was evaluated by Histone DNA ELISA assay **C.** Above experiments were also performed in stable HepG2 cells expressing dominant negative (T2609A) DNA-PKcs (dn-DNA-PKcs) or the empty vector (pSV2 neo) **D-F.** Primary human HCC cells (line-1/-2), transfected with scramble control (“Scr siRNA”) or DNA-PKcs siRNA (200 nM each), were treated with PBS or KU-0060648 (“KU”, 300 nM). After 72h culture, DNA-PKcs and tubulin expressions were tested by Western blotting **G.** Cell proliferation was tested by MTT assay **H.** Stable HepG2 cells expressing the empty vector (“Vector”) or wild-type DNA-PKcs (wt-DNA-PKcs-Flag) were subjected to Western blotting assay **I.** and cell proliferation assay **J.** and **K.** “Transfection” stands for Lipofectamine 2000 reagent alone. DNA-PKcs expression (vs. Tubulin) was quantified. Experiments in this figure were repeated three times, with similar results obtained. n=5 for each repeat. Bars stand for mean ± SD *p < 0.05.

Since KU-006064 is a recently-characterized DNA-PKcs inhibitor [12, 19, 20], next we tested whether DNA-PKcs was the primary target of KU-0060648 in HCC cells. Two non-overlapping shRNAs against DNA-PKcs (Seq-1 and Seq-2, see Methods) [18] were applied. Western blotting results in Figure 3A demonstrated that DNA-PKcs protein expression was dramatically downregulated in stable HepG2 cells expressing DNA-PKcs shRNAs (Seq-1 and Seq-2). Notably, HepG2 cell proliferation was also inhibited with DNA-PKcs stable knockdown (Figure 3B). On the other hand, cell apoptosis level was increased (Figure 3C). Significantly, KU-0060648 was still anti-proliferative and pro-apoptotic in the DNA-PKcs-silenced cells, although its activity in these cells was relatively weak (Figure 3B and 3C). Meanwhile, we showed that KU-0060648 was more potent than traditional DNA-PKcs inhibitors (NU-7441 [32] and NU-7026 [33]) in inhibiting HCC cells (Supplementary Figure S2A and 2B). Note that KU-0060648 was applied at 500 nM, while the NU-7441 and NU-7026 were utilized at 5 μM (Based on previous studies [10, 23, 32, 33]). More importantly, KU-0060648 could exert anti-proliferative activity in NU-7441/NU-7026-treated HCC cells (Supplementary Figure S2A and S2B). Next, we introduced a dominant negative (T2609A) DNA-PKcs (“dn-DNAPKcs”) [18] to HepG2 cells, and stable cells were established (Figure 3D). In these cells, p-DNA-PKcs (Thr 2609) was blocked (Figure 3D). Results demonstrated that stable HepG2 cells with dn-DNA-PKcs grew slower (Figure 3E), and showed spontaneous apoptosis activation (Figure 3F). Once again, in these DNA-PKcs-mutated HepG2 cells, KU-0060648 could still exert cytotoxic effects (Figure 3E and 3F).

Therefore, first we showed that DNA-PKcs inhibition (by adding NU-7441 and NU-7026), silence (by shRNAs), or mutation (T2609A) didn't reach to same degree of HepG2 cell inhibition as KU-0060648 did. Second, KU-0060648 was still anti-proliferative and pro-apoptotic in the DNA-PKcs-silenced/-mutated cells. Third, KU-0060648 could still inhibit HepG2 cells that were already treated with known DNA-PKcs inhibitors (NU-7441 and NU-7026). These results suggest that DNA-PKcs is first a vital molecule for HCC cell survival and proliferation. Second, it is an important yet not exclusive target of KU-0060648. DNA-PKcs-independent mechanisms should also play an important role in mediating KU-0060648's actions in HCC cells.

In primary human HCC cells, targeted-siRNA was applied to transiently knockdown DNA-PKcs (Figure 3G). DNA-PKcs siRNA knockdown inhibited primary HCC cell growth (Figure 3H). Similarly, KU-0060648 could still induce an anti-proliferative activity in DNA-PKcs-silenced primary cancer cells (Figure 3H). Based on above results, we would propose that DNA-PKcs overexpression could promote HCC cell proliferation. Using the method described, we established stable HepG2 cells expressing wild-type DNA-PKcs (wt-DNA-PKcs, Flag-tagged) (Figure 3I). Western blotting assay confirmed DNA-PKcs overexpression in the stable HepG2 cells (Figure 3I). Consequently, cell proliferation, tested by MTT assay (Figure 3J) and cell number counting (Figure 3K), was also enhanced. These results together indicate that DNA-PKcs is an important but not exclusive target of KU-0060648 in HCC cells.

### KU-0060648 inhibits PI3K-AKT-mTOR activation in HCC cells, independent of DNA-PKcs inhibition

**Figure 4:**
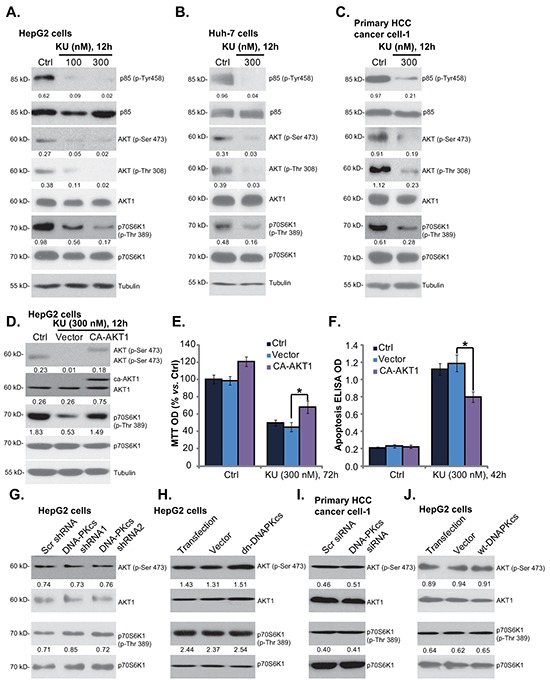
KU-0060648 inhibits PI3K-AKT-mTOR activation in HCC cells HepG2 **A.** Huh-7 **B.** and primary human HCC cells **C.** line-1) were treated with applied concentrations of KU-0060648 (“KU”) for indicated time, expressions of listed kinases and Tubulin were tested by Western blotting. Stable HepG2 cells expressing constitutively active AKT1 (CA-AKT1) or empty vector (“Ad-GFP”), were either left untreated (“Ctrl”), or treated with KU-0060648 (“KU”, 300 nM) for indicated time. Listed proteins were detected by Western blotting **D.** Cell proliferation **E.** and apoptosis **F.** were also tested. Expressions of listed kinases in HCC cells described in Figure 3 were tested **G-J.** Kinase phosphorylation (vs. total kinase) was quantified. Experiments in this figure were repeated three times, with similar results obtained. n=5 for each repeat (E and F). Bars stand for mean ± SD *p < 0.05.

Above results showed that KU-0060648 induced weak yet significant cell inhibition activity in DNA-PKcs-silenced/-mutated HCC cells, indicating that other mechanisms besides DNA-PKcs inhibition should be important for KU-0060648-exerted actions in HCC cells. ISt has been shown that KU-0060648 inhibits phosphatidylinositol 3-kinase (PI3K) and in-activates AKT-mTOR signaling [12]. We thus tested the PI3K-AKT-mTOR activation in KU-0060648-treated HCC cells. As demonstrated, KU-0060648 treatment significantly inhibited activation of PI3K (p85 phosphorylation), AKT (Ser-473 and Thr-308 phosphorylations) and mTOR (p70S6K1 Thr-389 phosphorylation) in both established (HepG2/Huh-7 lines) (Figure 4A and 4B) and primary human HCC cells (line-1, Figure 4C). Next, a constitutively-active AKT1 (CA-AKT1) [29] was introduced to HepG2 cells, and stable cells were selected. As demonstrated, CA-AKT1 restored activation of AKT and mTOR (p70S6K1 phosphorylation) in KU-0060648-treated cells (Figure 4D). More importantly, KU-0060648-induced proliferation inhibition (Figure 4E) and apoptosis (Figure 4F) were alleviated in CA-AKT1-expressing HepG2 cells. These results indicate that PI3K-AKT-mTOR inactivation should also participate in KU-0060648-exerted actions in HCC cells. Note that PI3K (p110 δ) expression was obviously higher in HCC cells than that in HL-7702 cells (Supplementary Figure S3A). Among the tested HCC cells, KYN-2 showed highest expression of PI3K p110 δ (Supplementary Figure S3A).

Next, we tested whether PI3K-AKT-mTOR inhibition is a secondary effect of DNA-PKcs inhibition. We showed that activation of AKT (Ser-473 phosphorylation) and mTOR (S6K1 phosphorylation) was intact in DNA-PKcs-silenced (Figure 4G) or DNA-PKcs-mutated (Figure 4H) HepG2 cells. Further, primary HCC cells with DNA-PKcs siRNA also showed equivalent AKT-mTOR activation, as compared to scramble siRNA-transfected HCC cells (Figure 4I). Meanwhile, AKT-mTOR activation was also unchanged in DNA-PKcs-overexpressed HepG2 cells (Figure 4J). These results indicate that PI3K-AKT-mTOR inhibition is likely a direct action by KU-0060648 in HCC cells, independent of DNA-PKcs inhibition.

### KU-0060648 suppresses HepG2 xenograft growth in nude mice

**Figure 5:**
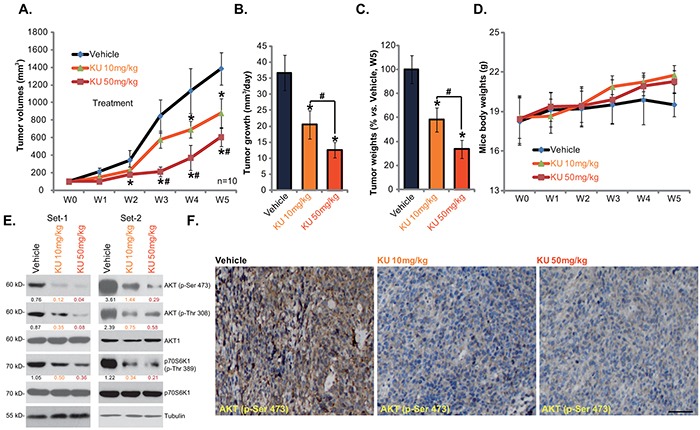
KU-0060648 suppresses HepG2 xenograft growth in nude mice HepG2 bearing nude mice (n=12 for each group: 10 for growth assay, 2 for signaling assay) were administrated with KU-0060648 (*i.p.* 10 and 50 mg/kg bodyweight, daily, for 21 days) or the vehicle control (saline), tumor volumes (in mm^3^) **A.** and mice body weights (in gram) **D.** were presented weekly, tumor daily growth was also calculated **B.** At the end of the experiments (week-5), tumors were separated through surgery and weighted **C.** Two weeks after initial KU-0060648 administration, two mice per group were sacrificed, HepG2 tumor tissues were isolated for Western blotting assay **E.** or IHC staining assay **F.** of indicated proteins, representative p-AKT (Ser473) IHC images were presented (Bar=50 μm) (F). Kinase phosphorylation (vs. total kinase) was quantified (E). Experiments in this figure were repeated twice, with similar results obtained. Bars stand for mean ± SD. “w” stands for week. *p < 0.05 vs. group “Vehicle”. ^#^*p* < 0.05 vs. KU-0060648 at 10 mg/kg group.

We also tested the *in vivo* activity of KU-0060648 using the HepG2 xenograft nude mice model. As described, a significant number of HepG2 cells were injected into the right flanks of nude mice, and xenografted tumors were established (Figure 5A). Administration of KU-0060648 (*i.p.* 10 and 50 mg/kg bodyweight, daily for 21 days) [12] dramatically inhibited HepG2 xenograft growth in nude mice (Figure 5A). Tumor daily growth in KU-0060648-admnistrated mice was significantly lower than that in vehicle control (saline) mice (Figure 5B). Further, the tumor weights (at week-5) of KU-0060648 group mice were also dramatically lighter than that of vehicle control mice (Figure 5C). Notably, KU-0060648 exerted a dose-dependent effect *in vivo*, KU-0060648 at 50 mg/kg was more potent than 10 mg/kg in inhibiting HepG2 xenografts (Figure 5A-5C). As shown in Figure 5D, mice body weights were almost not changed between each group in the tested durations, indicating that KU-0060648 administrations was generally safe to the experimental mice. We also failed to notice any deleterious side-effects in tested animals (vomiting, diarrhea, sudden weight loss, fever etc). Thus, we show that KU-0060648, at well-tolerated doses, suppresses HepG2 xenograft growth in nude mice.

We also tested the effect of KU-0060648 on AKT-mTOR activation *in vivo*. First, Western blotting was utilized to analyze signaling changes in above HepG2 xenografts (two mice per set). Results in Figure 5E showed clearly that administration of KU-0060648 dramatically inhibited AKT-mTOR activation in HepG2 xenografts. High-dose (50 mg/kg) of KU-0060648 was again more potent than low-dose (10 mg/kg) in inhibiting AKT-mTOR activation (Figure 5E). IHC staining results analyzing p-AKT Ser473 in Figure 5F (Set-1) further confirmed AKT inhibition by KU-0060648 administration, similar results were obtained in Set-2 (Data not shown). Thus, in line with the *in vitro* findings, *i.p.* administration of KU-0060648 inhibits AKT-mTOR activation in HepG2 xenografts.

### DNA-PKcs upregulation in human HCC cells and tissues, correlated with miRNA-101 downregulation

**Figure 6:**
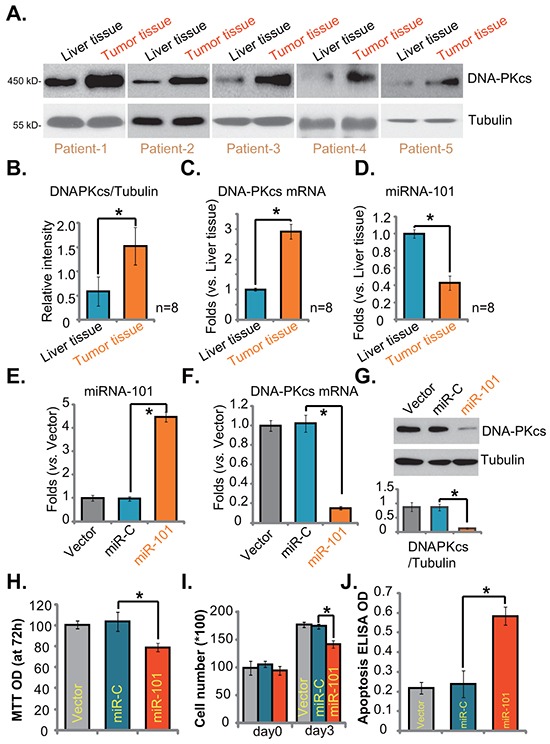
DNA-PKcs upregulation and miRNA-101 downregulation in human HCC cells and tissues DNA-PKcs (Protein and mRNA) and miRNA-101 (“miR-101”) expressions in surgery-isolated fresh human HCC tumor tissues (“Tumor tissues”) and surrounding normal liver tissues (“Liver tissues”) were shown **A, C.** and **D.** Protein expression of DNA-PKcs (vs. Tubulin) was quantified **B.** Stable HepG2 cells transfected with miR-101 construct, nonsense miRNA-control construct (“miR-C”), or the empty vector (pSuper-puro, “Vector”), were subjected to real-time PCR assay **E-F.** or Western blotting assay **G.** to test DNA-PKcs and miRNA-101 expressions. Proliferation **H.** and **I.** and apoptosis **J.** in above cells were also tested. Experiments in this figure were repeated three times, with similar results obtained. n=5 for each repeat. Bars stand for mean ± SD *p < 0.05.

Finally, we tested DNA-PKcs expression in human HCC tissues (“Tumor tissues”), and compared its level with surrounding normal liver tissues (“Liver tissues”). As demonstrated in Figure 6A, DNA-PKcs protein was overexpressed in HCC tissues (derived from eight different HCC patients). Its expression level was significantly higher in “Tumor tissues” vs. surrounding “Liver tissues” (Figure 6B). DNA-PKcs mRNA expression was also upregulated in HCC tissues (Figure 6C). We next studied the possible cause of DNA-PKcs upregulation in HCC by focusing on microRNAs (miRs). miRs are capable of regulating gene expression at translational or post-transcriptional levels [34, 35]. The 19-24 nucleotide single-stranded noncoding RNAs are shown to silence targeted mRNAs translation with partial complementarity in their 3′ untranslated regions (UTRs) [34, 35]. Existing evidences have shown that miR-101 could direct bind to and sequester DNA-PKcs mRNA [36]. Results in Figure 6D demonstrated clearly that miR-101 level in HCC tissues was significantly lower than that in surrounding normal liver tissues, which might be responsible for DNA-PKcs mRNA/protein upregulation in HCCs (Figure 6A and 6C).

Meanwhile, results in Supplementary Figure S3B and S3C demonstrated low miR-101 expression yet high DNA-PKcs mRNA expression in established or primary HCC cells, as compared to the non-cancerous HL-7702 cells. When we exogenously overexpressed miR-101 in HepG2 cells (Figure 6E), DNA-PKcs mRNA (Figure 6F) and protein (Figure 6G) expressions were correspondingly downregulated. As a result, HepG2 cell proliferation, tested by MTT assay (Figure 6H) and cell counting assay (Figure 6I), was inhibited. These miR-101-expressing HepG2 also showed spontaneous cell apoptosis (Figure 6J). As expected, nonsense miRNA-control construct (“miR-C”) showed no effect on DNA-PKcs expression or HepG2 cell proliferation (Figure 6E-6I). These results demonstrate DNA-PKcs overexpression in human HCC tissues, which is correlated with miRNA-101 downregulation.

## DISCUSSIONS AND CONCLUSIONS

The preclinical results of the current study indicate that KU-0060648, a novel DNA-PKcs inhibitor, could be a potential anti-HCC agent. First, expression of DNA-PKcs was upregulated in the tested human HCC tissues. Second, DNA-PKcs knockdown or mutation inhibited human HCC cell proliferation. Third, KU-0060648 exerted potent anti-proliferative and pro-apoptotic activities in established and primary human HCC cells. It was yet non-cytotoxic to human HL-7702 hepatocytes. Fourth, KU-0060648 suppressed PI3K-AKT-mTOR activation in human HCC cells. Fifth, intraperitoneal (*i.p.*) injection of KU-0060648 dramatically inhibited HepG2 xenograft growth in nude mice without causing apparent toxicities. Thus, DNA-PKcs is an important oncotarget for HCC [36, 37], and KU-0060648 might merit further investigations as a valuable anti-HCC agent.

We propose that the DNA-PKcs is an important but not exclusive target of KU-0060648 in HCC cells. HepG2 cells with DNA-PKcs silence (by shRNA/siRNA) or mutation displayed decreased proliferation and spontaneous apoptosis. Significantly, KU-0060648 was still anti-proliferative and pro-apoptotic in DNA-PKcs-silenced or -mutated cells, although its activity in these cells was relatively weak. Another important target by KU-0060648 in HCC cells could be PI3K-AKT-mTOR [12]. Overactivation of PI3K-AKT and its downstream mTORC1-S6K1 cascade is vital for HCC progression [38][3, 39-41]. Here we showed that KU-0060648 inhibited PI3K-AKT-mTOR activation in HCC cells. Reversely, CA-AKT1 restored AKT-mTOR activation, and inhibited KU-0060648-induced cytotoxicity in HepG2 cells. These results indicate that PI3K-AKT-mTOR in-activation is involved in KU-0060648-exerted actions in HCC cells.

As described by Munck et al [12], KU-0060648 is an ATP-competitive PI3K inhibitor, which targeted all subunits of PI3K (α, β, λ, and ψ) [12]. KU-0060648 showed highest potency against the PI3K p110 δ subunit [12]. AKT-mTOR inhibition was the result of PI3K blockage by KU-0060648 [12]. We first showed that activation of PI3K, tested by p-p85 and p-AKT, was inhibited by KU-0060648 in HCC cells. Second, we showed that PI3K p110 δ expression was higher in HCC cells than that in HL-7702 cells (Supplementary Figure S3A). Among the tested HCC cells, KYN-2 showed highest expression of PI3K p110 δ (Supplementary Figure S3A). High PI3K p110 δ expression and high DNA-PKcs expression (Supplementary Figure S3C) in KYN-2 cells might explain the superior sensitivity of these cells to KU-0060648 (Figure 1D, lowest IC-50).

Existing evidences have implied that DNA-PKcs may directly activate AKT-mTOR. For example, Feng and co-authors showed that DNA-PKcs formed a complex with AKT, leading to a 10-fold increase of AKT activity [23]. Dragoi and colleagues demonstrated that activated DNA-PKcs could act as an AKT kinase [42]. Ji et al., displayed that Ultra Violet (UV) radiation induced DNA-PKcs association with mTORC2 component Sin1 to phosphorylate AKT at Ser-473 [10]. In the current study, however, we indicate that PI3K-AKT-mTOR inactivation is unlikely a downstream event of DNA-PKcs inhibition. AKT-mTOR activation was intact in DNA-PKcs-silenced/-overexpressed or DNA-PKcs-mutated HepG2 cells. Significantly, KU-0060648 was still anti-proliferative and pro-apoptotic in DNA-PKcs-inhibited/-silenced/-mutated HCC cells. Thus, PI3K-AKT-mTOR inhibition could be a direct action by KU-0060648 in HCC cells. This might also explain the superior activity of KU-0060648 in HCC cells, more potently than traditional DNA-PKcs inhibitors (NU-7026, NU-7441).

For many years, HCC has been otherwise a chemo-resistant malignancy [3, 4]. Molecularly-targeted therapy could be a extremely important option for HCC [3, 4]. Our results show that KU-0060648 potently inhibits HCC cells *in vitro* and *in vivo*, indicating a possible therapeutic value for HCC.

## MATERIALS AND METHODS

### Culture of established cell lines

Human HCC cell lines, including HepG2, Huh-7 and KYN-2, as well as human HL-7702 hepatocytes were purchased from the Cell Bank of CAS Shanghai (Shanghai, China) at Dec 2014. Cells were maintained in RPMI medium, supplemented with 10% fetal bovine serum (FBS), penicillin/streptomycin, in a humidified incubator. All cell culture reagents were provided by Gibco Life Technologies (Carlsbad, CA). The cell line verification was described in Supplementary Information.

### Isolation of human HCC tissues, and culture of primary cells

Surgery-isolated primary human HCC tissues were washed in DMEM. Tumor tissues and surrounding normal liver tissues were separated very carefully under microscopy. A total of eight different primary HCC patients, administered in Wuxi People's Hospital (Wuxi, China), were included in the study (All male, 42-64 years old). These patients received no chemotherapy or radiotherapy prior to surgeries. Fresh tissues were stored in liquid nitrogen. For primary culture of HCC cells, cancer tissues were subjected to collagenase I (Sigma) digestion for 30 min. The resolved single-cell suspensions were then pelleted, washed, and re-suspended in primary cell culture medium (DMEM, 20%-FBS, 2 mM glutamine, 1 mM pyruvate, 10 mM HEPES, 100 units/mL penicillin/streptomycin, 0.1 mg/mL gentamicin, and 2 g/liter fungizone) [21]. Experiments and protocols requiring human samples were approved by the Internal Review Board (IRB) of all authors' institutions. The written-informed consent was obtained from each participant. All studies using human samples were conducted according to the principles expressed in the Declaration of Helsinki and according to national and international guidelines.

### Chemicals, reagents and antibodies

KU-0060648 was provided by GuideChem (Shanghai, China). The caspase-3 specific inhibitor z-DEVD-fmk and the general caspase inhibitor Z-VAD-fmk were purchased from Sigma Chemicals (Louis, MO). NU-7026 and NU-7441 were purchased from Calbiochem (San Diego, CA). p-DNA-PKcs (Thr 2609) antibody was purchased from Santa Cruz (Shanghai, China), All other antibodies utilized in this study were obtained from Cell Signaling Tech (Danvers, MA), as described previously [10, 22]. The concentrations of agents applied and the treatment durations were chosen based on published literatures and results from pre-experiments.

### MTT assay of cell proliferation

Cell proliferation was determined by 3-(4,5-dimethylthiazol-2-yl)-2,5-diphenyltetrazolium bromide (MTT) assay as described [21].

### DNA-PKcs siRNA knockdown

DNA-PKcs siRNA composed of two targeted-sequences, 5′-AGGGCCAAGCTGTCACTCT-3′ [23] and 5′-GAUCGCACCUUACUCUGUUTT-3′ [14], was synthesized by Genechem (Shanghai, China). siRNA (200 nM) transfection was performed through Lipofectamine 2000 (Invitrogen, Karlsruhe, Germany) following the manufacturer's instructions [24]. Expression of DNA-PKcs in transfected cells was verified by Western blotting.

### DNA-PKcs shRNA and stable cell selection

Two non-overlappingDNA-PKcs shRNA lentiviral GV248 plasmids were provided by Dr. Han [18]. These two sets of DNA-PKcs lentiviral-shRNAs were named as DNA-PKcs shRNA Seq-1 and DNA-PKcs shRNA Seq-2. The lentiviral-shRNAs directly added to cultured HepG2 cells (with 60% of confluence, cultured in serum-free medium). After 12 h, virus-containing medium was replaced with fresh complete medium. Stable HepG2 colonies were selected by puromycin (5 μg/mL, Sigma) for 10-12 days. Expression of DNA-PKcs in stable cells was verified by Western blotting.

### DNA-PKcs mutation or overexpression

As described [10], the 3-kb HindIII fragment of DNA-PKcs cDNA covering Thr-2609 (Genechem) was utilized as the template for generating the DNA-PKcs T2609A cDNA. The T2609A DNA-PKcs pSV2 neo Flag plasmid [10] or the wild-type (wt-) DNA-PKcs pSV2 neo Flag plasmid (gift from Dr. Li He at Kunming Medical University) [10] was transfected into HepG2 cells with the Lipofectamine 2000 protocol (Invitrogen) [10]. After 48 h, HepG2 cells were re-plated on selection medium containing 100 μg/mL of G418 for 10 days. Stable colonies were isolated, and characterized for expression of DNA-PKcs (Flag-tagged).

### RNA extraction and real-time PCR

As previously reported [24, 25], total RNA was prepared through TRIzol reagent (Invitrogen). Real Time-PCR assay was performed. Briefly, 1 μL of diluted reverse transcription (RT) product was utilized as template. The PCR reaction mixture contains 1× SYBR Master Mix (Applied Biosystem, Foster City, CA), 500 ng RNA and 200 nM primers. An ABI Prism 7500 Fast Real-Time PCR system (Foster City, CA) was used for PCR reactions. The following primers were used: *GAPDH*, forward: 5′-TGC ACC ACC AAC TGC TTA-3′; reverse: 5′-GGA TGC AGG GAT GAT GTT C-3′ [26]. miR-101: forward: 5′-CGG CGG TAC AGT ACT GTG ATA A-3′, reverse: 5′-CTG GTG TCG TGG AGT CGG CAA TTC-3′ (Universal stem-loop primer) [24, 27]. DNA-PKcs, forward, 5′-CCT GCG GGT AGT TAT CAG TGA TTT-3′, and reverse, 5′-CCA CTT ACA GGA TCA TAG CGA CGA ATG C-3′ [28]. After amplification, melt curve analysis was performed to analyze product melting temperature. *GAPDH* gene was chosen as the reference gene for normalization, and the 2^−ΔΔ*Ct*^ method was applied to quantify targeted mRNA change within samples [24, 25].

### microRNA (miRNA) transfection

miRNA-101 (miR-101) expression pSuper-puro construct was described in our previous study [24]. Non-sense miRNA-control (“miR-C”) was purchased from Ambion (Shanghai, China). For transfection, HepG2 cells were seeded on to six-well plates at 50% confluence. Transfection was performed through Lipofectamine 2000 transfection reagent (Invitrogen) (0.15 μg construct per transfection). Twelve h after transfection, cell medium was replaced with 2 mL of complete medium containing 2% FBS. Puromycin (5.0 μg/mL, Sigma) was then added to establish stable cells (10-14 days), which were always checked for miR-101 and DNA-PKcs expressions.

### Constitutively active-AKT1 (CA-AKT1) expression and stable cell line selection

CA-AKT1 vector and the empty vector (Ad-GFP) were reported in our previous study [29]. The plasmid (0.10 μg/mL) was transfected into HepG2 cells with the Lipofectamine 2000 protocol [29]. Stable cells were selected by puromycin (5.0 μg/mL) for 10-12 days. Western blotting was utilized to verify AKT expression/activation in stable cells.

### *In vivo* anti-tumor efficiency assay

A significant amount of HepG2 cells (5 millions/mice) were injected subcutaneously into the right flanks of female nude mice (6-8 weeks old). When tumors reached around 100 mm^3^, mice were randomized into three groups with 12 mice per group: vehicle control (saline), 10 mg/kg of KU-0060648 (intraperitoneal injection or *i.p.*, daily, for 21 days), and 50 mg/kg of KU-0060648 (*i.p.*, daily, for 21 days) [12]. The injection was started when the tumors were established (volumes around 100 mm^3^). Tumor volumes, recorded every week, were calculated through the established formula: Volume (mm^3^) = (*d*^2^ × *D*)/2, in which d and D were the shortest and the longest diameter, respectively. Two weeks after initial KU-0060648 administration, xenografted tumors of two mice per group were isolated, and were subjected to Western blotting and immunohistochemistry (IHC) staining assays. Humane endpoints were applied to minimize suffering. Five weeks after initial KU-0060648 administration, HepG2 xenografts were separated through surgery and weighted. All studies were performed in accordance with the standards of ethical treatment approved by the Institutional Animal Care and Use Committee (IACUC) and Association for the Assessment and Accreditation of Laboratory Animal Care (AAALAC). The protocols of the in vivo study were approved by the Animal Care and Use Committee at all authors institutions.

### Immunohistochemistry (IHC) staining

As described [21, 24], the staining was performed on cryostat sections (3 μm) of xenograft tissues (two mice per group set) according to standard methods. We incubated slides in the appropriate dilutions of primary antibody (anti-p-AKT Ser473, 1:50) and subsequently stained them with horseradish peroxidase (HRP)-coupled secondary antibody (Santa Cruz). We visualized peroxidase activity using 3-amino-9-ethyl-carbazol (AEC) and counterstained tissues with MAYER's solution (Merck).

### For primary culture of human peripheral blood mononuclear cells (PBMCs) and human skin fibroblasts (HSFs), “Clonogenicity” assay, [H^3^] Thymidine incorporation assay of cell proliferation, Caspase-3 activity assay, Histone DNA-ELISA assay of cell apoptosis and Western blotting

please refer to our previous studies [21, 25, 29-31]. These methods were described in detail in the Supplementary Information.

### Statistical analysis

Data were presented as mean ± standard deviation (SD). Statistics were analyzed by one-way ANOVA followed by a Scheffe' and Tukey Test (SPSS 15.0). Significance was chosen as *p* < 0.05. IC-50 was calculated by the SPSS software.

## SUPPLEMENTARY INFORMATION FIGURES


