# Supplementary material for: KU-0060648 inhibits hepatocellular carcinoma cells through DNA-PKcs-dependent and DNA-PKcs-independent mechanisms
Source: Oncotarget. 2016 Feb 26;7(13):17047–59. doi: 10.18632/oncotarget.7742 (PMC4941370; doi:10.18632/oncotarget.7742)
Supplement: Supplementary file 1 [file oncotarget-07-17047-s001.pdf]

SUPPLEMENTARY INFORMATION

Cell line confirmation

DNA fingerprinting and profiling were performed every 3 months to confirm the origin of the cell line, and to distinguish the cell line from cross-contamination. All cell lines were subjected to mycoplasma and microbial contamination examination every month. Population doubling time, colony forming efficiency, and morphology under phase contrast were also measured every 3 months under defined conditions to confirm the phonotype of cell line.

Primary culture of human peripheral blood mononuclear cells (PBMCs) or human skin fibroblasts(HSFs)

PBMCs of informed-consent healthy donors (male, 25-35 years old) were isolated by centrifugation with the lymphocyte separation medium (Sigma). After three washes in PBS, PBMCs were counted and cultured in PBMC primary culture medium as described previously [1]. Primary human skin fibroblasts (HSFs), obtained from the Cell Bank of Fudan University (Shanghai, China), were maintained in DMEM medium plus 10% FBS.

[H^3] Thymidine incorporation assay of cell proliferation

Cells were treated with applied concentration of KU-0060648 plus 1 µCi/ml of tritiated thymidine. To determine [H^3] thymidine incorporation, cells were washed with cold PBS, the DNA was precipitated with cold 10% trichloroacetic acid (TCA), solubilized with 1.0 M sodium hydroxide, and aliquots were counted by liquid-scintillation spectrometry. The value of treatment group was normalized to that of untreated control group.

Clonogenicity assay

To determine colony formation, HCC cells (0.2 × 106) were seeded onto the 6-well tissue culture plate, allowed to attach for 24 h and treated with applied treatment. Then, cells (400 cells/well) were re-seeded onto another 6-well tissue culture plate, and cultured for additional 8 days. Afterwards, colonies were stained with crystal violet solution and counted.

Caspase-3 activity assay

Following the indicated treatment/s, cytosolic proteins of approximately 1 × 106 cells were extracted. Thirty µg of cytosolic extracts per sample were added to caspase assay buffer (312.5 mm HEPES, pH 7.5, 31.25% sucrose, 0.3125% CHAPS) with benzyloxycarbonyl-DEVD-7-AFC (amido-4-(trifluoromethyl)coumarin) (10 µg/mL) as the substrate (Promega, Madison, WI). After 1 h of incubation at 37 °C, the release of AFC was quantified, using a Fluoroskan system (Thermo-Labsystems, Helsinki, Finland) with an excitation value of 355 nm and emission value of 525 nm.

Quantification of apoptosis by ELISA

The Cell Apoptosis Detection ELISA Kit (Roche, Shanghai, China) was applied to quantify cell apoptosis according to the attached protocol. Briefly, cell lysates (20 µg proteins in 20 µL lysis buffer/sample) were overlaid and incubated in the 96-well microtiter plate modules coated with anti-histone antibody (Roche). Samples were then incubated with anti-DNA peroxidase for 10 min at room temperature under the dark, followed by color development with ABTS substrate. The absorbance of the samples was determined with a microplate reader (Tecan, Durham, NC) at 405 nm.

Western blotting

As previously described [2], aliquots of 30 ±g of lysed proteins from each cell or tissue sample were separated by SDS polyacrylamide gel, and transferred onto polyvinylidene difluoride (PVDF) membranes (Millipore, Shanghai, China). Following blocking (10% milk in PBST for 1 h), membranes were incubated with specific antibodies overnight at 4 °C. Goat anti-mouse IgG or goat anti-rabbit IgG horseradish peroxidase (Santa Cruz Biotechnology) were utilized as secondary antibodies. Enhanced chemiluminescence (ECL) was applied for detection (Amersham Bioscience, Freiburg, Germany). Representative blots of at least three independent experiments were shown. The intensity of each band was quantified by ImageJ software. All image processing in this study was performed by Photoshop software (7.0).

REFERENCES

1. Yang L, Weng W, Sun ZX, Fu XJ, Ma J and Zhuang WF. SphK1 inhibitor II (SKI-II) inhibits acute myelogenous leukemia cell growth in vitro and in vivo. Biochem Biophys Res Commun. 2015; 460:903-908.

2. Chen MB, Jiang Q, Liu YY, Zhang Y, He BS, Wei MX, Lu JW, Ji Y and Lu PH. C6 ceramide dramatically increases vincristine sensitivity both in vivo and in vitro, involving AMP-activated protein kinase-p53 signaling. Carcinogenesis. 2015; 36:1061-1070.

SUPPLEMENTARY FIGURES

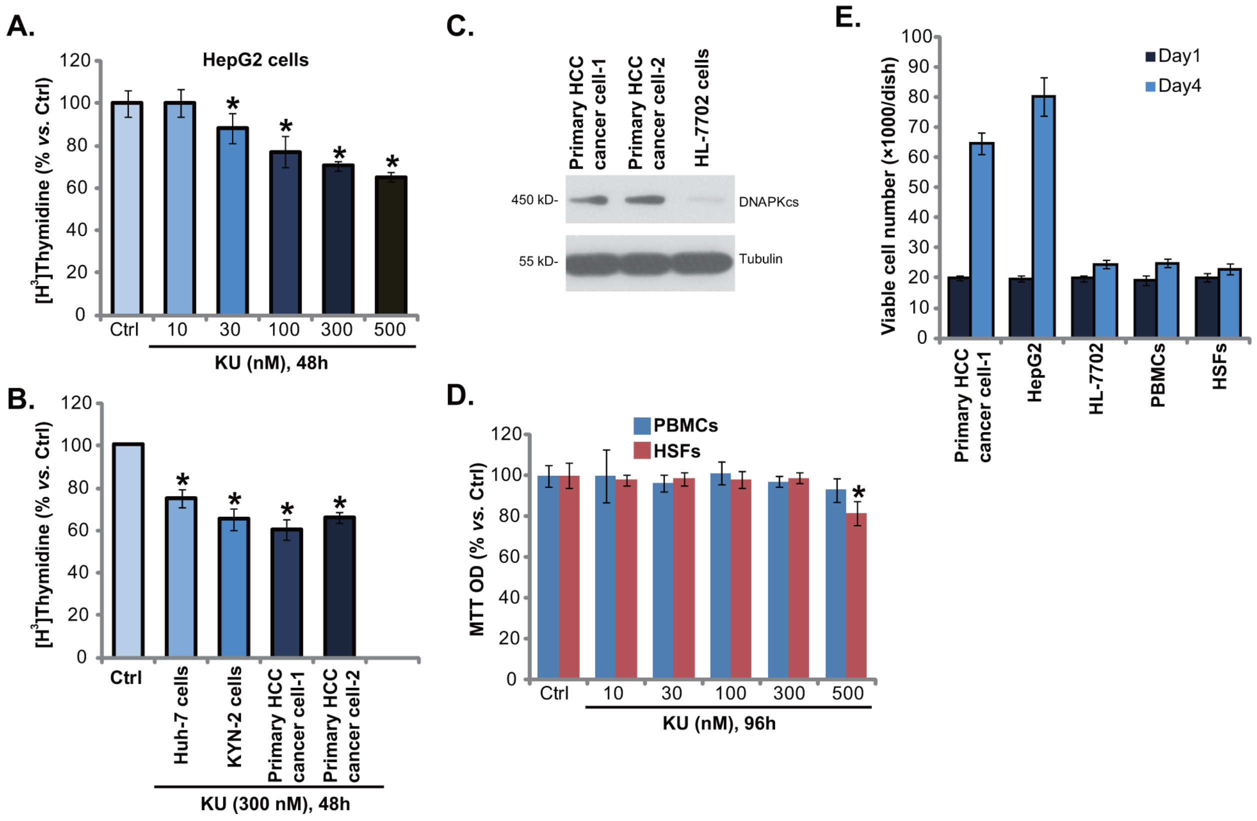

Supplementary Figure S1: HepG2, Huh-7 and KYN-2 cells or the primary human HCC cells (line-1/-2), as well as peripheral blood mononuclear cells (PBMCs) and primary human skin fibroblasts (HSFs) were either left untreated, or treated with applied KU-0060648 ("KU"), cells were then cultured for indicated time. Cell proliferation was tested by $[H^3]$ Thymidine incorporation assay **A.** and **B.** or MTT assay **D.** DNA-PKcs and tubulin expressions in primary human HCC cells (line-1/-2) and in HL-7702 human hepatocytes were shown **C.** The viable (trypan blue exclusive) cell number of listed cells at day 1 and day 4 was shown **E.** Experiments in this figure were repeated four times, with similar results obtained. n=5 for each repeat. Bars stand for mean ± SD * ***p*** < 0.05 vs. group "Ctrl".

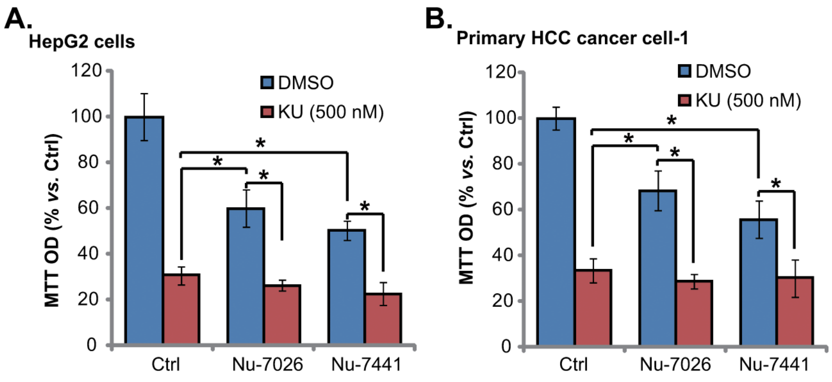

Supplementary Figure S2: HepG2 A. or primary human HCC cells **B.** line-1) were treated with KU-0060648 ("KU", 500 nM) and/or NU-7441/NU-7026 (5 µM each), cell proliferation was tested by MTT assay. Experiments in this figure were repeated four times, with similar results obtained. n=5 for each repeat. Bars stand for mean ± SD. "DMSO" stands for 0.1% DMSO. * ***p*** < 0.05.

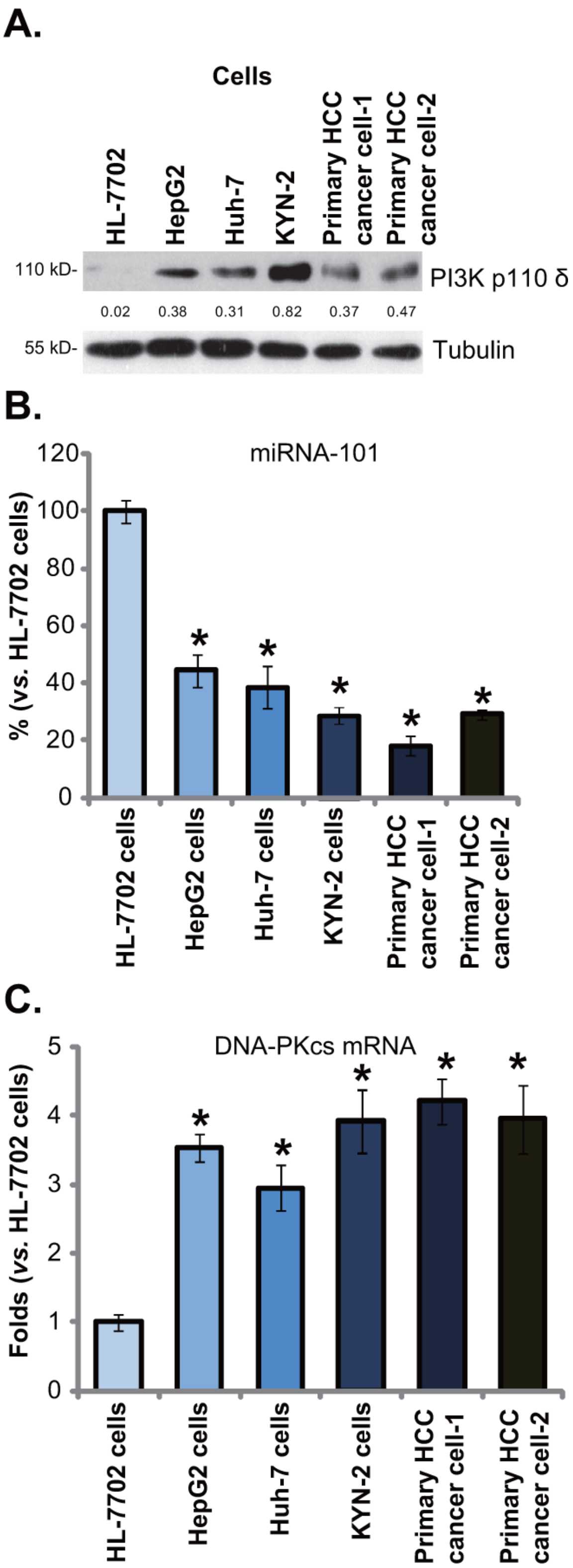

Supplementary Figure S3: PI3K p110 δ and Tubulin protein A. miRNA-101 ("miR-101") **B.** DNA-PKcs mRNA **C.** expressions in the listed HCC cells or HL-7702 cells were shown. Experiments in this figure were repeated three times, with similar results obtained. n=5 for each repeat. PI3K p110 δ expression (vs. Tubulin was quantified). Bars stand for mean ± SD ***p** < 0.05 vs. HL-7702 cells.
